# Ascites and Its Treatment

**Published:** 1865-12

**Authors:** William Baker

**Affiliations:** Howden


### ASCITES AND ITS TREATMENT.

BY WILLIAM BAKER, M. A., M. D.

Dropsies undoubtedly arise from a great variety of causes. They are frequently the mere consequence of disease in remote parts. Probably any obstruction to the circulation, whatever that may be, and whether in the large viscera, the veins, the lymphatics, may be productive of asceties; this we term symtomatic dropsy, and our success will depend upon the nature and site of the obstruction. There are many cases of ascities which we are justified in calling and treating as idiopathic. The exact causes of such dropsies is certainly not clearly indicated, but I should say it is strictly local: the heart, lungs, liver, kidneys, &c., appear perfect in structure and in function; and it is probable that the effusion is caused by some morbid condition of the peritoneum. But in the cases I

have lately witnessed I think that condition was not inflammation; there were no febrile symptoms, no tenderness or pain; the general health was not bad: the mechanical effect of the effused fluid might be blamed for all the discomfort that was experienced. The balance in secretion and absorption in dropsies is certainly destroyed, the exhalents are undoubtedly acting in excess, and the cause may be vital or structural. I have presumed it to be the latter, and strictly local, and therefore have placed much confidence in local medication, which I strongly recommend in addition to all other indicated means.

Three cases of what I will call chronic idiopathic ascites have lately fallen to my care—all strong, not unhealthy looking men, from fifty-five to sixty years of age. Two of them had led active and regular lives; the other, Mr. W——, had occasionally indulged, and committed errors in both eating and drinking: I will give some particulars of his case.

Mr. W——, aged sixty, had been aware of the gradually increasing size of his abdomen and its nature for somemonths; and in January, 1864, I found the effusion sufficiently large to have sanctioned tapping. Seven years before he had been under my care in a similar condition, and been perfectly unloaded by medical means alone; therefore, on this occasion, the former treatment was recurred to. It consisted of hydragogues, salines, with iodine, bitters, &c.; friction to the abdomen, and continued pressure; the hip-bath, of a grateful temperature, every other night. The local applications used in succession were tincture of digitalis, variously combined, mercurial liniment, and ointment of iodide of potassium.

The above means were most diligently used for three months. The hydragogues acted most abundantly, frequently unloading him to the extent of four, five and six quarts in the twenty-four hours; yet the accumulation was not lessened. In April, thinking I might gain some advantage to my treatment by tapping, four gallons of dirty-red, watery fluid were abstracted. For three weeks after tapping there was no perceptible disposition to the return of the dropsy; but in a few days more the accumulation was again evident, and, in spite of the most vigorous use of all my means, it uninteruptedly progressed. In July the distension was so great as to require a second operation, and four gallons and two pints of the same dirty-red looking fluid were removed. In a few days after this second tapping the effusion rather rapidly returned; yet the patient was comfortable, without the most trifling fever. Blisters were applied to the abdomen, the salines, the hydragogues,

&c., continued; and so soon as he had recovered from the blisters, the local application and pressure were resumed.

This plan was steadily persisted in. The hydragogues were pushed to the most warranted extent till September, when it was ascertained by the *tape* that the effusion had increased. The salines, with iodide of potassium and hydragogues, were continued, and tincture of iodine was substituted for the former local applications, the abdomen painted with it every night, the bandage to be used of every comfortable degree of tightness, and the bath as before. In October the tape showed a trifling diminution of size; the means were continued, and in November the advantage was still more evident. Every week now told in favor of the treatment, and during December the effusion was completely removed. On the 1st of January, 1865, an occasional painting was recommended, and a course of salines, aperients, and bitters to be continued for some time. Mr. W—— is now well. The other two cases, in much less time, yielded to similar treatment.

Three cases of success are certainly too scanty to establish confidence in any particular plan of medical treatment where the *post hoc* and the *propter hoc* are so difficult to define. I know not if the tincture of iodine has been used by others in the same manner. At the present time I certainly attach much importance to it and deem it worthy of trial. The plan of treatment in Mr. W——'s case was little changed throughout; the hydragogues were probably pushed to a greater extent before the painting with the tincture of iodine than after; yet it is clear that no advantage was gained over the effusion until the painting had been continued for some time, and then the reduction was so slow, but uninterruptedly progressive under its use, that I certainly am inclined to attribute the principal benefit to it. In the treatment of dropsies, the most powerful means we possess are undoubtedly hydragogues. When the strength of the patient will allow, and there is nothing to contraindicate their continued use, we can generally succeed in unloading the patient, and in rendering him at least a temporary good. The best hydragogues are colocynth with croton oil, gamboge, jalap, and cream of tartar, or elaterium and colomel variously combined. For continued use there is nothing equal to, nor as manageable as, elaterium. My dose of elaterium is one-twentieth of a grain. With this dose I invariably commence, and rarely find it necessary to carry it beyond one-tenth of a grain, in combination with minute doses of calomel, and squill pill, to which the compound genitan mixture with sulphate of magnesia will be found a most efficient adjuvant, and may be taken with the elaterium pill every four, six or eight hours. In urgent cases of cellular dropsy, where the powers of life are almost overwhelmed by the effusion, I certainly prefer gamboge, jalap and cream of tartar. With three doses of this compound, one every four hours, I unloaded a patient of Mr. Newstead, of Budwith, of twelve quarts in the twenty-four hours.

This was a most urgent case, consequent upon taking cold, with serious organic lesion of the heart and lungs, and the hydragogues were required to be repeated twice. after suitable intervals of repose, for the full removal of the effused fluid, the result of the second administration was seven quarts; in the aggregate six gallons.

Howden, Sept. 1865.

